# Shoulder Movement-Centered Measurement and Estimation Scheme for Underarm-Throwing Motions

**DOI:** 10.3390/s24102972

**Published:** 2024-05-07

**Authors:** Geunho Lee, Yusuke Hayakawa, Takuya Watanabe, Yasuhiro Bonkobara

**Affiliations:** Graduate School of Engineering, University of Miyazaki, 1-1 Gakuen-Kibanadai Nishi, Miyazaki 889-2192, Japan; hi19034@student.miyazaki-u.ac.jp (Y.H.); hi20050@student.miyazaki-u.ac.jp (T.W.)

**Keywords:** boccia, underarm throw, throwing estimation, shoulder movement, launch angle, regression analysis

## Abstract

Underarm throwing motions are crucial in various sports, including boccia. Unlike healthy players, people with profound weakness, spasticity, athetosis, or deformity in the upper limbs may struggle or find it difficult to control their hands to hold or release a ball using their fingers at the proper timing. To help them, our study aims to understand underarm throwing motions. We start by defining the throwing intention in terms of the launch angle of a ball, which goes hand-in-hand with the timing for releasing the ball. Then, an appropriate part of the body is determined in order to estimate ball-throwing intention based on the swinging motion. Furthermore, the geometric relationship between the movements of the body part and the release angle is investigated by involving multiple subjects. Based on the confirmed correlation, a calibration-and-estimation model that considers individual differences is proposed. The proposed model consists of calibration and estimation modules. To begin, as the calibration module is performed, individual prediction states for each subject are updated online. Then, in the estimation module, the throwing intention is estimated employing the updated prediction. To verify the effectiveness of the model, extensive experiments were conducted with seven subjects. In detail, two evaluation directions were set: (1) how many balls need to be thrown in advance to achieve sufficient accuracy; and (2) whether the model can reach sufficient accuracy despite individual differences. From the evaluation tests, by throwing 20 balls in advance, the model could account for individual differences in the throwing estimation. Consequently, the effectiveness of the model was confirmed when focusing on the movements of the shoulder in the human body during underarm throwing. In the near future, we expect the model to expand the means of supporting disabled people with ball-throwing disabilities.

## 1. Introduction

Throwing motions are fundamental movement skills and a basic ability that people should acquire [1,2,3]. People use the throwing motion for rehabilitation activities, contributing to the maintenance of mental and physical health [4,5,6]. In other words, this motion improves athletic ability and has a positive psychological impact. However, people with profound weakness, spasticity, athetosis, or deformity in the upper limbs struggle to control their hands to hold or release a ball using their fingers with proper timing. Typical aids for them include hand orthoses [7,8,9] and prosthetic hands [10,11,12]. Although people intend these aids for daily life activities, they are not suitable for assisting throws that require fine or agile coordination [13,14].

Boccia is a sports event designed for people with serious functional disabilities, and has recently become an official event at the Paralympic Games. The rules of boccia resemble those of curling, with players competing to determine who can get a ball closer to a target by throwing. Recently, boccia has been spreading as a recreational pastime due to the simplicity of its rules and its high usefulness. It has become a familiar ballgame for everyone from very young children to elderly people [15,16]. Although players use throws in boccia matches, those who have difficulty throwing can also participate. These players may kick balls or use an assistive device such as a ramp. Quite a few people with difficulty in pitching would like to participate in games using their own throwing motions.

In rare instances, a boccia player with hand disorders may throw by wearing a small pocket on the back of the hand to hold a ball. Several players throw the ball using an assistive device designed to the level of their individual symptoms [17,18]. However, this method does not solve the fundamental problem of releasing the ball with proper timing. Moreover, assistive devices are not versatile, and depend on the level of disorder [19,20]. On the other hand, recent studies on throwing motion estimation based on contactless sensors such as motion capture systems have been introduced [21,22,23]. This enables highly accurate estimation for the throwing motions of the entire body; however, not only does it require a large amount of data, it limits the estimation environment. Unlike existing studies, we seek another assistive approach that utilizes throwing motions and caters to as many players as possible regardless of individual differences. Unfortunately, no such approaches have been found yet.

Thus, this paper presents an estimation scheme for throwing motion intention as a first step towards providing throw support. Specifically, it considers underarm throwing, a useful pose in boccia. Based on input data collected during the throwing motion, estimation parameters related to release timing are examined. Next, the generality of the input data used for estimation needs to be ensured. For this purpose, we examine which part of the body to measure and how to connect the measured body part to the throwing intention. Accordingly, this paper aims to find an assistive clue by understanding throwing motions. We expect this to greatly increase the number of methods that can use and support the throwing action.

The rest of this paper is organized as follows. Section 2 introduces the problem and its definition. Section 3 details the formulation of the underarm throwing motions. Section 4 proposes an enhanced estimation scheme based on the formulation of the underarm throwing motion and presents the evaluation results from extensive experiments to verify its effectiveness. In addition, this section presents our findings and future directions. Finally, Section 5 concludes our paper.

## 2. Problem Statement

### 2.1. Considerations of Underarm Throwing

This paper addresses what to measure during a ball throw and how to estimate from the measured motions. When it comes to ball-throwing actions, we need to consider the following three conditions: (1) throwing style, (2) the body part used for measurement and estimation, and (3) the estimation parameters. Among these conditions, we first consider the throwing style. Boccia players largely adopt two types of throwing styles: overarm and underarm throws [24,25]. More players prefer the underarm throw, as it offers a tactical advantage [26,27]. Consequently, the underarm throw style is more suitable for finding clues to use their tactical throws.

Second, we select the shoulder as the body part to observe for estimation from the standpoint of information acquisition. Many studies have focused on the kinematics of throwing motions [28,29], and the shoulder plays an important role in acquiring information about them [30,31]. In practice, a number of studies have attempted to use shoulder motion as an interface for prosthetic hands. This trial indicates that the shoulder provides a large amount of motion data [32,33].

Third, we discuss the estimation parameters for throwing. When people throw toward a desired location with an underarm style, they tactically select a more appropriate speed and launch angle [34,35]. We believe that by estimating the speed and launch angle, we can then guess the location of a ball after it is thrown. Although throwing actions such as the swinging speed of the arm affect the ball speed, the timing of releasing the ball strongly influences the launch angle of the ball.

### 2.2. Problem Definition

Based on the above considerations, this study defines the “throwing intention” as the launch angle when underarm throwing. Next, shoulder movements as input signals for estimation are employed. Additionally, assuming that a throwing intention will be introduced into assistive devices, we aim to propose an estimation scheme that accounts for contactless sensors and can be used in various places.

**Figure 1:**
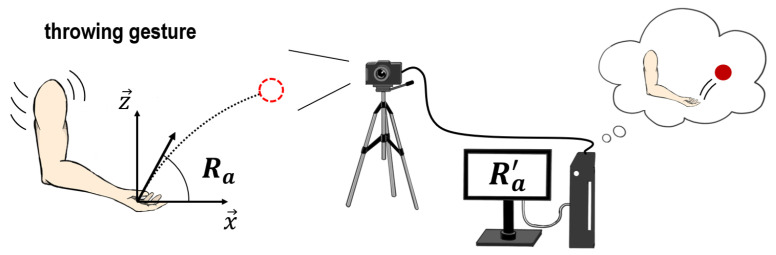
Conceptual illustration of measurement and estimation for underarm throwing motions. The launch angle when a swing is conducted is defined as $R_{a}$, while the estimated launch angle after recording by a measurement device such as a camera is represented by $R_{a}^{\prime }$.

The objective of this paper is to determine how to estimate the motional intentions when underarm throwing. As our solution approach, we aim to estimate the launch angle from the swing of an arm during underarm throwing. As illustrated in Figure 1, the launch angle when a swing is conducted is defined as $R_{a}$. Moreover, the estimated launch angle after recording by a measurement device such as a camera is represented by $R_{a}^{\prime }$. Depending on the definitions, the relationship between $R_{a}$ and $R_{a}^{\prime }$ is described as

(1) $$R_{a}=R_{a}^{\prime }.$$

When tackling the problem, Equation (1) indicates the solution direction of how to estimate $R_{a}$ from the swing of the arm. We attempt to accomplish this study using the following procedure:

- Select an appropriate body part, enabling the estimation of $R_{a}$.
- Investigate the relationship between the movements of the selected part and $R_{a}$ and evaluate its generality.
- Build an estimation model using the obtained relation that is adjusted for individual differences and demonstrate its effectiveness through evaluation tests.

## 3. Formulation of Underarm Throwing Motion

### 3.1. Observation of Shoulder Movements

The shoulder was determined as the part of the body, as it is the closest part of the upper limbs to the trunk of the body. Furthermore, the trunk and upper limbs greatly contribute to the ball-throwing motion, and they are connected by the shoulder.

When a ball is thrown in the underarm throwing style, an arm (including a hand) is located below a shoulder [36]. Acromion movements in the shoulder are clearly observed during underarm throwing. In other words, there are few blind spots hidden by the underarm throwing motion. For these reasons, the movements of the acromion are regarded as representative motions of the shoulder. For convenience, the movements of the acromion were measured. Moreover, the geometric relationship between the shoulder’s motions and $R_{a}$ was investigated through preliminary experiments and analyses.

### 3.2. Definition of Ball-Throwing Motions

**Figure 2:**
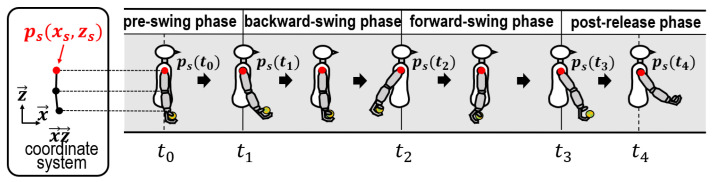
Classification of four phases according to swing motion during underarm throwing.

To analyze the underarm throwing motion efficiently, we first described the throwing motion before conducting preliminary experiments. As shown in Figure 2, a series of underarm throwing motions was divided into four phases according to the state of the swinging arm and the condition of ball holding. First, the period from holding the ball to before swinging the arm backward was called the pre-swing phase. Second, the period in which the arm is swung backward was called the backward-swing phase. Third, the period when the arm swings forward before releasing the ball was defined as the forward-swing phase. Fourth, the period after releasing the ball was called the post-release phase. The times required for each phase are represented by $t_{1}$, $t_{2}$, $t_{3}$, and $t_{4}$, respectively.

A substantial disparity in motions is presumed during the pre-swing and post-release phases among various people; thus, these phases were excluded from our analytical process. Our analyses focused on the motions in the backward-swing and forward-swing phases, which are expressed as $B_{sp}$ and $F_{sp}$, respectively. Based on the sagittal plane, the throwing direction is set to $\vec{x}$. The axis $\vec{z}$ is defined by rotating $\vec{x}$90° and counterclockwise. The origin intersects $\vec{x}$ with $\vec{z}$. This defined coordinate system is expressed as $\vec{x}\vec{z}$*-coord* for convenience. The typical coordinates of the shoulder are the acromia coordinates, symbolized as $p_{s}$$\left(=\left(x_{s},z_{s}\right)\right)$ (see Figure 2) with respect to the $\vec{x}\vec{z}$*-coord*.

### 3.3. Experimental Settings for Measurement of Underarm Throwing Motion

**Figure 3:**
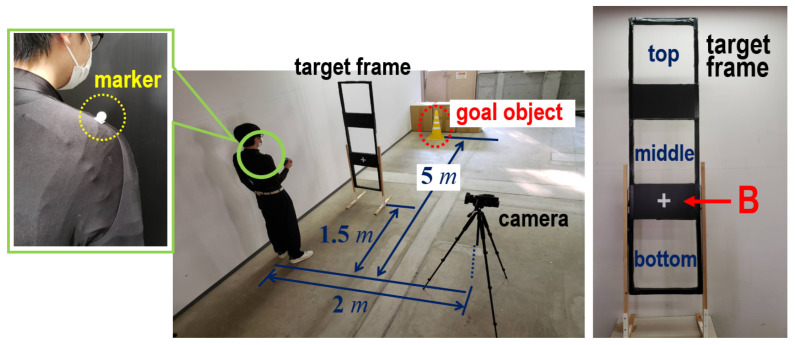
Preparation for preliminary experiments to investigate the relationship between $p_{s}$ and $R_{a}$. The target frame was turned upright and was positioned in front of each subject. Three target plates (the top, middle, and bottom) in the target frame were installed from the top downward. To capture $p_{s}$ in line with the changes in $R_{a}$, clothes that fit the individual subjects were used. An in-house marker was attached to the shoulder of the subject. To adjust each height of the three plates to the height of individual subjects, the height of a marker “B” and the level of the hand when standing upright were matched.

Figure 3 shows the equipment used in the experiments and its settings. To make a distinction of $R_{a}$, the target frame was turned upright and positioned in front of each subject. Three target plates (the top, middle, and bottom) in the target frame were installed from the top downward. To capture $p_{s}$ in line with the changes in $R_{a}$, clothes that fit the individual subjects were used. An in-house marker was attached to the shoulder of the subject (left side of Figure 3). To curtail any influence on data acquisition due to marker slippage, the marker location was checked and the clothes were adjusted after pitching. The FDR-AX700 digital 4K video camera recorder (SONY Corporation, Tokyo, Japan) with 1080p and 120 fps was placed beside the subject to capture the movement of the marker. We intended to suppress the disparity in the throwing speed by explaining the throwing method so that the ball could fall near the goal object.

As mentioned in Section 2, measurement data from multiple subjects are required when investigating the relationship between $p_{s}$ and $R_{a}$. Through open recruitment within our university, seventeen subjects who agreed to our experimental plan participated (fourteen males and three females, with a mean age of 23 ± 2 years old, height 155∼188 cm). Before the preliminary experiments, the permission of the ethics committee of the University of Miyazaki was obtained (Approval No.: 2021-001). Furthermore, the informed consent of all subjects was obtained after providing them with a sufficient explanation of the experiments.

The reasons why we determined to use the FDR-AX700 video camera in the preliminary experiments were as follows. Employing a CMOS sensor type, this video camera with a total of 14.2 megapixels provides a 16:9 aspect ratio. This wide-screen format results in throw analyses that are effective for obtaining a broad range of movements. The camera’s installed BIONZ X image processor allowed accurate image processing to be obtained for the throwing motions.

Through the above settings, the experiments were performed as follows: (1) the subjects wore fitted clothes and pasted the marker on their shoulder; (2) each height of the three plates was adjusted following the height of individual subjects (the height of a marker “B” and the level of the hand when standing upright were matched on the right side in Figure 3); and (3) the subjects threw 10 balls for each of the top, middle, and bottom plates, for a total of 30 balls).

### 3.4. Experimental Results for the Measurement of Underarm Throwing Motion

Two types of data were obtained from the experiments. The method for acquiring these data is described below. The first data consisted of the $R_{a}$ measurements when the ball was thrown on the three plates (top, middle, and bottom). $R_{a}$ was collected from the captured video clips using a sampling frequency of 120 Hz. In practice, the data of $R_{a}$ tracking each ball were based on the use of the KINOVEA analysis software (Kinovea-0.9.4-x64). We computed $R_{a}$ as the mean angle of the ball (based on $\vec{x}$) within 50 ms of the release. The data for each of the 30 throws by individual subjects were obtained by excluding data that could not be properly extracted due to poor camera focus. Next, within $B_{sp}$ and $F_{sp}$, the second data were $p_{s}\left(t\right)$ according to time. $p_{s}\left(t\right)$ was obtained by tracking the movements of the marker on the captured data.

**Figure 4:**
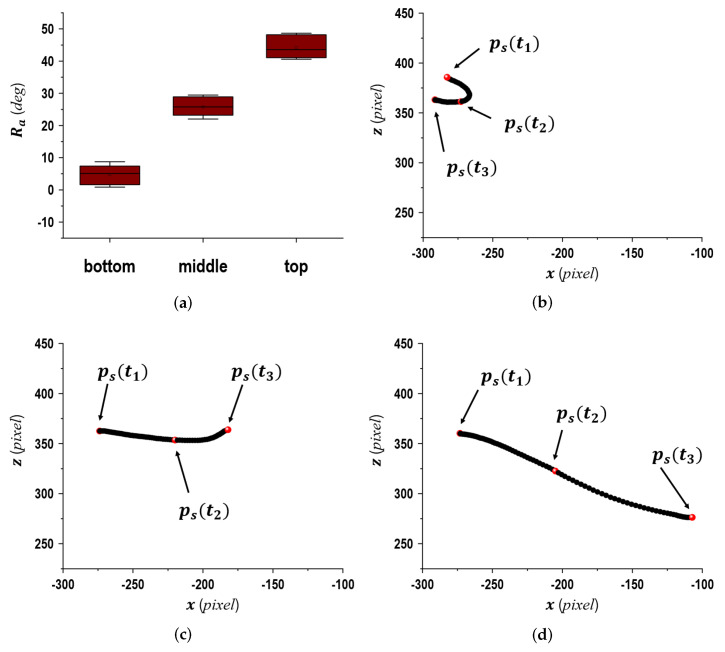
Experimental results for $R_{a}$ (upper left) and displacement variations of $p_{s}$ with respect to $\vec{x}\vec{z}$*-coord* when the balls were thrown in each plate (the top, middle, and bottom) of the target frame, as shown in Figure 3. (**a**) $R_{a}$, (**b**) top plate, (**c**) middle plate, (**d**) bottom plate.

Figure 4 displays $R_{a}$ at the time when 10 balls were thrown by one subject in each plate (the top, middle, and bottom), as well as $p_{s}\left(t\right)$ for one of the 10 balls thrown. From the results in Figure 4a, we validated that $R_{a}$ presented a low disparity. However, it changed slightly depending on the plates. Additionally, the data in Figure 4b–d demonstrate different movements for the two phases of $B_{sp}$ and $F_{sp}$ following the changes in $R_{a}$.

**Figure 5:**
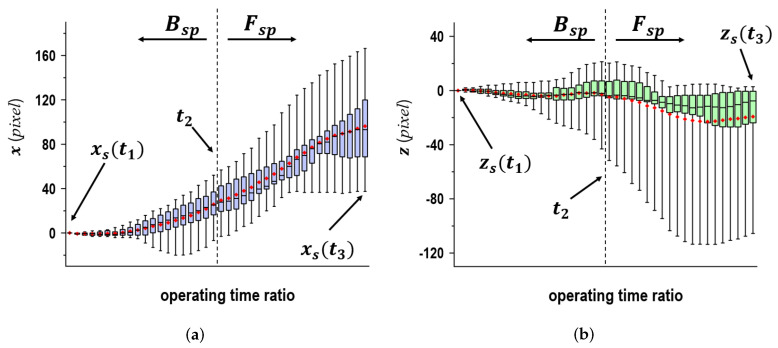
Shoulder movements in $\vec{x}$ and $\vec{z}$ in time ratio for seventeen subjects; the values of *x* and *z* in $t_{1}$ were used as the reference. (**a**) $\vec{x}$ direction, (**b**) $\vec{z}$ direction.

Figure 5 presents the displacement quantity of $p_{s}$ in the $\vec{x}$ and $\vec{z}$ directions as the time ratio when individual subjects threw 10 balls in the middle plate. Here, the error bars indicate 95% confidence intervals and the boxes represent distributions of measured data in the range of 25–75%. Within these boxes, the black bars and the red dots symbolize the medians and averages according to the time ratio, respectively. It can be observed that despite throwing the ball into the same plate, $p_{s}\left(t\right)$ varied not only with each subject but also with each ball that was thrown. A similar trend was observed for displacement. Hardly any change was observed in the $\vec{z}$ direction relative to the movement in equivalent intervals in the positive $\vec{x}$ direction in relation to the time ratio at the $B_{sp}$ phase. Therefore, a linear movement in relation to $p_{s}\left(t\right)$ with respect to the $\vec{x}\vec{z}$*-coord* shows a similar trend. As for $F_{sp}$ which we validated in the $\vec{x}$ direction, the type of movement was the same as that in $B_{sp}$. In the $\vec{z}$ direction, the variations ranged from the negative direction to the positive direction. A curved movement was noted in relation to $p_{s}\left(t\right)$.

**Figure 6:**
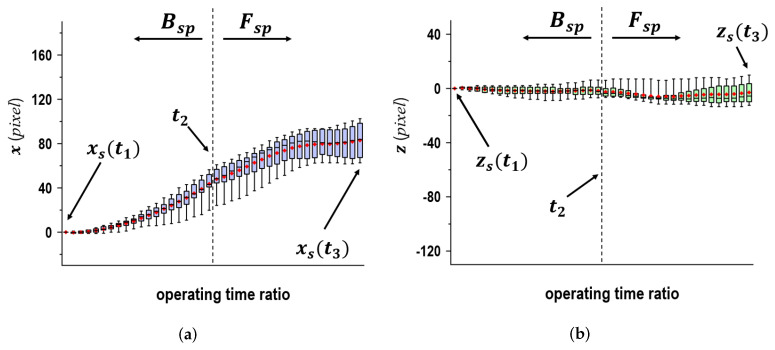
Shoulder movements in $\vec{x}$ and $\vec{z}$ in time ratio for one subject; where the values of *x* and *z* in $t_{1}$ were used as the reference. (**a**) $\vec{x}$ direction, (**b**) $\vec{z}$ direction.

In Figure 5, the two graphs depict wide variations in the error bars when comparing the ranges of individual boxes with them. Although this may be due to individual differences, these results had a large range of variations in the error bars. To ensure familiarity with the experimental settings, throwing practices before the measurements were conducted with one subject. In detail, we had the subject throw ten balls toward each target plate. Figure 6 shows the displacement quantity of $p_{s}$ in the $\vec{x}$ and $\vec{z}$ directions as the time ratio when the subject threw 10 balls in the middle plate. In spite of the similar trends in Figure 5, it was verified that the variations in the error bars were reduced. Pretraining on throwing could be an effective way to maintain a constant posture, resulting in reduced fluctuations in the data.

After the completion of the experiments, the seventeen subjects were asked to fill out a questionnaire. The questions were as follows:

- Have you been joining in recreational activities that involve throwing a ball (such as boccia)?
- Do you find upper extremity movements, including throwing movements, burdensome?

For the first question, four respondents (23.5%) said yes, eleven (64.7%) said no, and two (11.8%) had no response. The four subjects who replied in the affirmative said that they participated in baseball games more than 2–3 times a month. For the second question, all subjects answered no.

### 3.5. Model of Underarm-Throwing Motion

**Figure 7:**
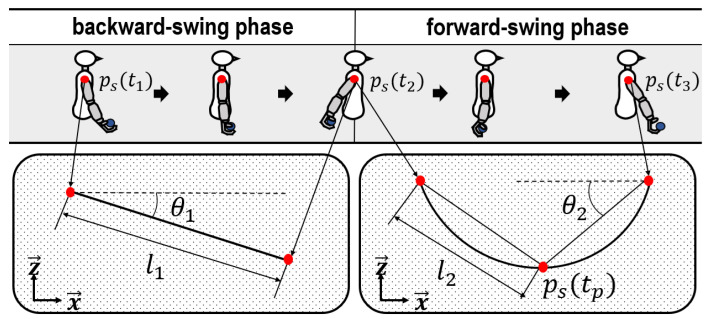
Illustration for movement trend of $p_{s}\left(t\right)$ in underarm throwing. To observe $p_{s}\left(t\right)$ in practice, a marker was attached to the shoulder, as shown in Figure 3.

It was confirmed that there was a presence of constant tendencies in $p_{s}\left(t\right)$. Based on the results, these tendencies fluctuated in accordance with $R_{a}$. To simplify the analyses, $p_{s}\left(t\right)$ was modeled. $p_{s}\left(t\right)$ was derived and its fluctuations were observed by the change of $R_{a}$ into a variable. The modeling of $p_{s}\left(t\right)$ within $B_{sp}$ is presented as a linear model based on the trends depicted on the left side of Figure 7. Next, $F_{sp}$ is represented as a curved model on the right side of Figure 7. In the results compared in Figure 4, fluctuations occur in $p_{s}\left(t\right)$ following the changes in $R_{a}$. Therefore, $p_{s}\left(t\right)$, in which fluctuation is notably observed in the aforementioned model, was set as a variable.

The variables described in Figure 7 are explained below. For $B_{sp}$, a significant change was acquired in the movement quantity $l_{1}$ when the shoulder moves in the positive $\vec{x}$ and on the slope $\theta_{1}$. Based on the conceptual description, $l_{1}$ and $\theta_{1}$ can be expressed as follows:

(2) $$l_{1}=\sqrt{{\left(x_{s}\left(t_{2}\right)-x_{s}\left(t_{1}\right)\right)}^{2}+{\left(z_{s}\left(t_{2}\right)-z_{s}\left(t_{1}\right)\right)}^{2}},$$

(3) $$\theta_{1}=\tan^{-1}\left(\frac{z_{s}\left(t_{2}\right)-z_{s}\left(t_{1}\right)}{x_{s}\left(t_{2}\right)-x_{s}\left(t_{1}\right)}\right).$$

For $F_{sp}$, a significant change is observed in the movement quantity $l_{2}$ during the movement in the negative $\vec{z}$ and on the slope $\theta_{2}$ before the release of the ball. Given the minimum value of $p_{s}\left(t_{p}\right)$ in the curved model, if it is based on $p_{s}\left(t_{3}\right)$ from the release to 50 ms before the release, then $l_{2}$ and $\theta_{2}$ can be provided as follows:

(4) $$l_{2}=\sqrt{{\left(x_{s}\left(t_{p}\right)-x_{s}\left(t_{2}\right)\right)}^{2}+{\left(z_{s}\left(t_{p}\right)-z_{s}\left(t_{2}\right)\right)}^{2}},$$

(5) $$\theta_{2}=\tan^{-1}\left(\frac{z_{s}\left(t_{p}\right)-z_{s}\left(t_{2}\right)}{x_{s}\left(t_{p}\right)-x_{s}\left(t_{2}\right)}\right).$$

### 3.6. Discussion of Underarm Throwing Motion Model

In this subsection, the impact of these variables on $R_{a}$ according to the time ratio in each phase is divided and analyzed. A well-known regression equation is employed when $R_{a}$ is the objective variable for the analysis method. Additionally, $l_{1}$ in Equation (2) and $\theta_{1}$ in Equation (3) are used as the explanatory variables of $B_{sp}$. In $F_{sp}$, the explanatory variables are defined as $l_{2}$ and $\theta_{2}$ in Equations (4) and (5), respectively. The determination coefficient of $R^{2}$ is employed to indicate how well the regression equation and actual data fit for the evaluation method. As the analysis deals with quantitative data, the actual $R_{a}$ is used when throwing the ball toward the three plates. The regression equation of $B_{sp}$ is as follows:

(6) $$R_{a}=a_{1}l_{1}+a_{2}\theta_{1}+a_{0},$$

where $a_{1}$ and $a_{2}$ denote regression coefficients and $a_{0}$ indicates a fragment. Next, the regression equation of $F_{sp}$ is

(7) $$R_{a}=b_{1}l_{2}+b_{2}\theta_{2}+b_{0},$$

where $b_{1}$ and $b_{2}$ refer to the regression coefficients and $b_{0}$ to the fragment.

**Figure 8:**
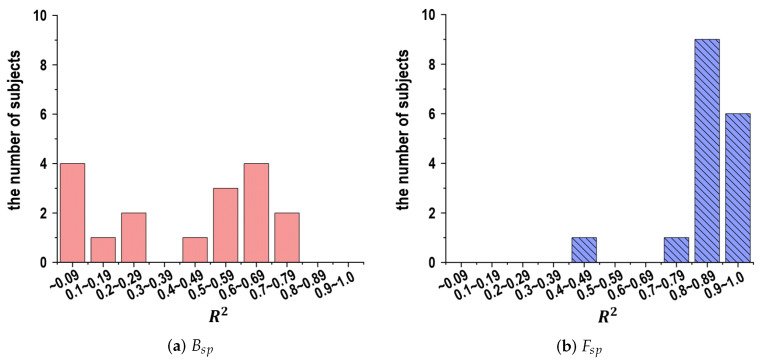
Analytical results for the number of subjects corresponding to the determination coefficient of $R^{2}$ in Equations (6) and (7) according to $B_{sp}$ and $F_{sp}$. (**a**) $B_{sp}$, (**b**) $F_{sp}$.

Figure 8a,b shows the analytical results for the number of subjects corresponding to the determination coefficient of $R^{2}$ in Equations (6) and (7) according to $B_{sp}$ and $F_{sp}$, respectively. Here, the vertical directions indicate the number of subjects in relation to the determination coefficients. In Figure 8a, although some subjects present a highly precise correlation with $R_{a}$, the results exhibit obvious disparity depending on the subject. On the other hand, in Figure 8b the variables in $F_{sp}$ present a highly precise correlation with $R_{a}$ for many subjects.

**Figure 9:**
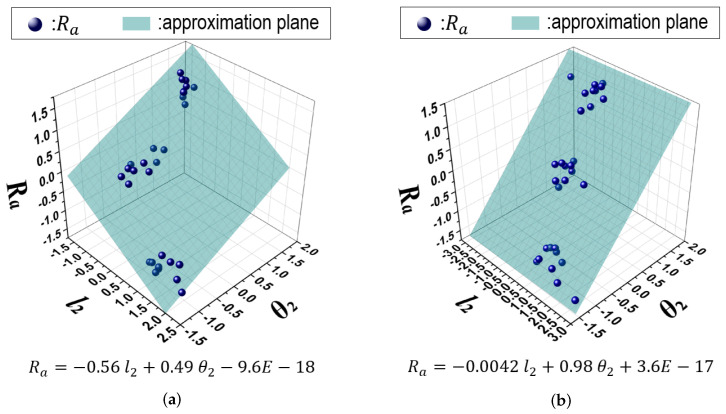
Analytical results for $l_{2}$ and $\theta_{2}$ corresponding to $R^{2}$ in Equation (7) according to two subjects. (**a**) subject #1 ($R^{2}$: 0.905), (**b**) subject #2 ($R^{2}$: 0.946).

To validate a highly precise correlation for $F_{sp}$, we plotted $R_{a}$ in relation to $l_{2}$ and $\theta_{2}$ in Figure 9. These results indicate that the actual data accumulated around the regression equation, namely, the approximation plane. From the results, a highly precise correlation was visually confirmed. Moreover, the slope of the correlation (coefficients and a fragment in the regression equation) varied among the subjects. We considered the main causes that contributed to the results to be individual differences such as ball-throwing poses and the rotational range of the shoulder joints.

**Figure 10:**
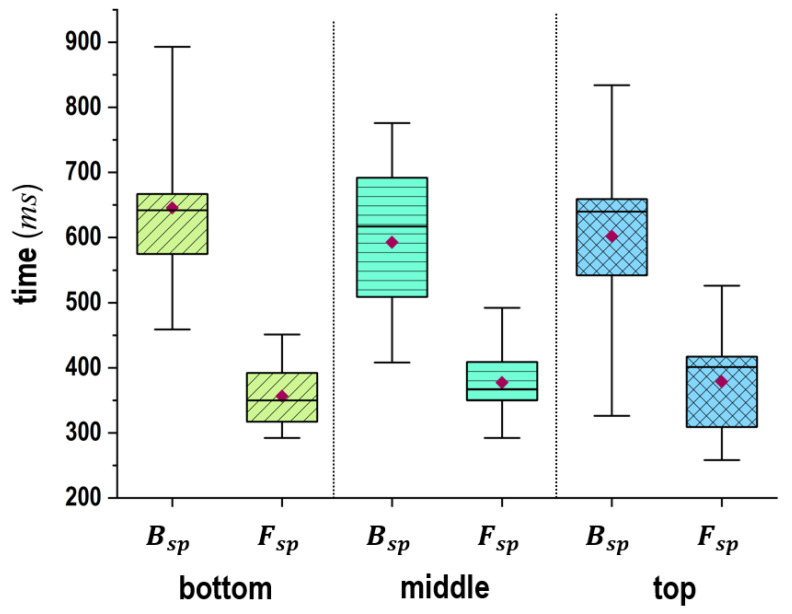
Analytical results of the required times for $B_{sp}\left(t_{1}∼t_{2}\right)$ and $F_{sp}\left(t_{2}∼t_{3}\right)$ when 10 balls were thrown to each plate (the top, middle, bottom) in the target frame by seventeen subjects.

Finally, Figure 10 confirms that the time required for $F_{sp}$ is shorter than for $B_{sp}$ and that the results for $F_{sp}$ have a slighter variance depending on the subjects. Specifically, the subjects requiring 500 to 700 ms for $B_{sp}$ and 300 to 400 ms for $F_{sp}$ had a majority. Moreover, $t_{1}∼t_{2}$ and $t_{2}∼t_{3}$ had a slight effect on the changes in the target plates. Based on these results, it could be concluded that $F_{sp}$ requires less time and has less variation among subjects compared to $B_{sp}$.

## 4. Enhanced Estimation Scheme and Evaluation Results

From the results of the preliminary experiments in Section 3, we confirmed the highly precise correlation of $p_{s}\left(t\right)$ with respect to $F_{sp}$ with $R_{a}$. However, $p_{s}\left(t\right)$ was obtained offline by tracking the movements of the marker on the captured data. Furthermore, the slope of this correlation varied among subjects. To overcome these limitations, this section introduces an enhanced online model for estimating $R_{a}$ that takes individual differences into account. We demonstrate the effectiveness of the model through evaluation tests while considering previous results.

### 4.1. Calibration and Estimation Model for Underarm Throwing Motions

**Figure 11:**
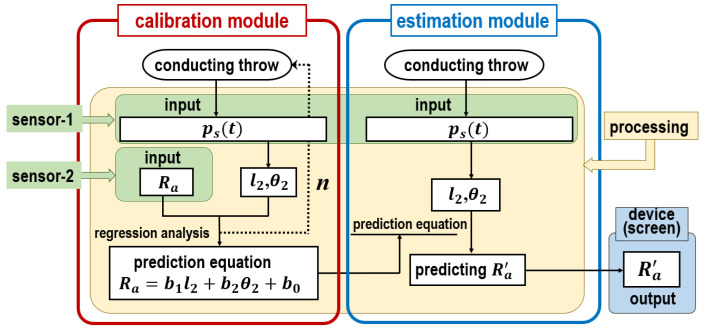
Computation process of the calibration and estimation model for underarm throwing motions.

As an enhanced estimation scheme, a calibration and estimation model for underarm throwing motions is proposed. The computation flow in this model is shown in Figure 11. The model comprises calibration and estimation modules. First, the prediction equation for each subject is updated as the calibration module is performed. Then, $R_{a}^{\prime }$ is estimated employing the updated prediction equation in the estimation module.

In practice, in order to perform an online process during underarm throwing it is necessary to refer $p_{s}\left(t\right)$ and $R_{a}$ simultaneously. For that purpose, sensor-1 and sensor-2 are respectively employed to observe $p_{s}\left(t\right)$ and $R_{a}$ in real time. The details of the computational processing of the model by the use of sensor-1 and sensor-2 are explained below.

To begin, the calibration module updates the slope of the correlation varying with the subjects. In detail, during ball throwing, $p_{s}\left(t\right)$ is obtained using sensor-1. Then, $l_{2}$ and $\theta_{2}$ are sought from the data. Meanwhile, $R_{a}$ is input from sensor-2. By repeating the process *n* times, the regression analysis is performed using these variables and the prediction equation for individual subjects is derived accordingly. This equation is the same as the regression equation (see Equation (7)), and uses the correlation confirmed in $F_{sp}$.

Next, $R_{a}^{\prime }$ is estimated from $p_{s}\left(t\right)$ by executing the estimation module. The prediction equation refined by the calibration module is applied to predict $R_{a}^{\prime }$, then $l_{2}$ and $\theta_{2}$ are obtained in a manner similar to that of the calibration module during ball throwing. As $R_{a}$ is actually thrown by the subject, it is acquired by substituting $l_{2}$ and $\theta_{2}$ obtained in the prediction equation. The estimation module outputs $R_{a}^{\prime }$ that was actually thrown as $R_{a}$.

The calibration module copes with individual differences in ball-throwing styles as the highlighting point in the model. A feature emerges as the generality of the motion estimation, as no restrictions and complexity are imposed on specific programs executing the computation processing on sensor-1 and sensor-2 extracting the data and on the device outputting the results. However, the reliability of the proposed model depends on the *n* number of balls thrown in the calibration module. Therefore, it is necessary to evaluate the dependence between the *n* number of balls thrown and the estimation accuracy.

### 4.2. Evaluation Experiments for Calibration and Estimation Model

**Figure 12:**
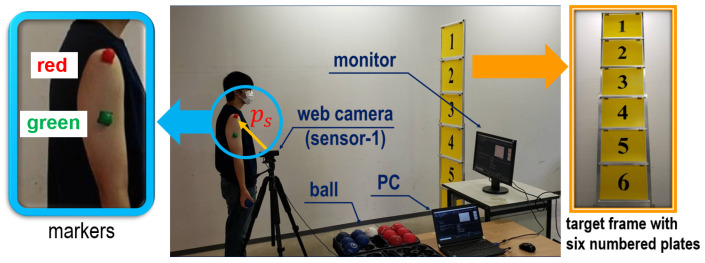
Preparation for evaluation experiments.

We performed evaluation experiments for the calibration and estimation model with seven subjects to verify its effectiveness. Figure 12 shows the experimental settings. A target frame with six numbered plates was prepared to examine the estimation accuracy of $R_{a}^{\prime }$. We used a C920R web camera (Logicool Co., Ltd., Tokyo, Japan) with 1080p and 30 fps as sensor-1. In practice, the use of the web camera allowed to online compute $p_{s}\left(t\right)$ on an LG Gram laptop PC (LG Electronics Co., Ltd., Seoul, Republic of Korea) with an Intel(R) Core(TM) Ultra 7 processor 155H (Intel, Santa Clara, CA, USA) without transferring the motion data captured by the video camera. Moreover, an LG 27UL500-W monitor (LG Electronics Co., Ltd., Seoul, Republic of Korea) was used to display $R_{a}^{\prime }$ based on $p_{s}\left(t\right)$ computed in the model.

Next, the calibration module in the proposed model was used to update the prediction equation online while conducting throws. Red and green markers were used to clearly extract $p_{s}\left(t\right)$ with respect to $F_{sp}$ from the motion data captured by sensor-1. As shown in Figure 12, these markers were attached to the shoulder and upper arm of each subject, respectively. Similar to the preliminary experiments in Section 3, $p_{s}\left(t\right)$ was read with the red marker. On the other hand, the green marker was used to differentiate the beginning time of $F_{sp}$ from the overall swing motion. After the time of $F_{sp}$ was determined, $l_{2}$ and $\theta_{2}$ were computed and applied to the online prediction equation (see the calibration module in Figure 11). In practice, the extraction of these data was implemented using a Python-oriented real-time object tracking technique through a web camera. Finally, boccia balls were used for the throw.

**Figure 13:**
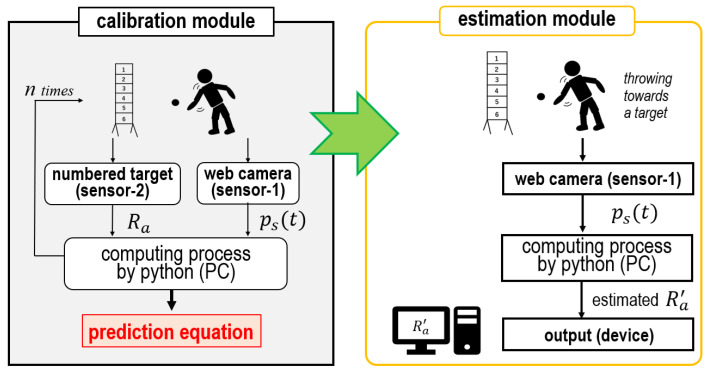
Process flow of the calibration and estimation model implemented as experimental settings.

Figure 13 presents the procedure for the evaluation experiments. Details on the procedure are as follows. (1) The subject throws a ball toward a plate identified in advance. Simultaneously, the main controller records $l_{2}$ and $\theta_{2}$ extracted from $p_{s}\left(t\right)$ and the plate number where the plate is hit. (2) The subject repeats the first procedure *n* times. The main controller computes the prediction equation by performing regression analysis based on the saved data. (3) After calibration, the subject throws a ball at a plate and the main controller then estimates the numbered plate based on the prediction equation. In the evaluation experiments, the output of the prediction equation should display Arabic numerals rounded down after the decimal point as the estimated number on the monitor, as the number of targets is $R_{a}^{\prime }$.

We evaluated the estimation accuracy through these experiments based on the number of hits when 20 balls were thrown toward the target plates. Two evaluation directions were set to demonstrate the effectiveness of the proposed model: (1) how many balls needed to be thrown in advance to generate the prediction equation with sufficient accuracy (*n* numbers of throws in the calibration module), and (2) whether the proposed model could reach sufficient accuracy despite individual differences. Based on these directions, the following experiments were conducted:

- Investigating the estimation accuracy after changing the number of balls *n* thrown in the calibration module
- Performing the evaluation experiments with multiple subjects and evaluating the estimation accuracy based these results.

### 4.3. Evaluation Results and Discussion

**Figure 14:**
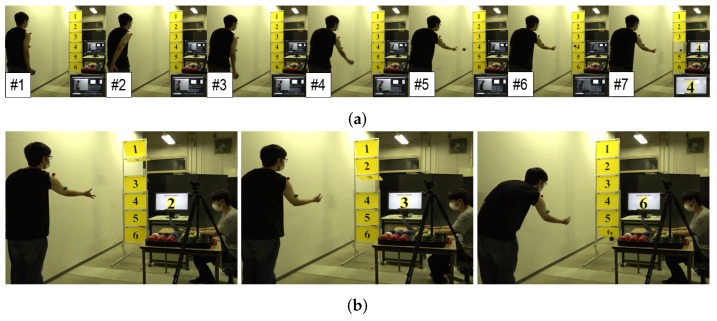
Experimental scenes used to investigate estimation accuracy. (**a**) a series of underarm-throwing motions toward the four numbered plate, (**b**) underarm-throwing motion toward the two, three, and six numbered plates.

Figure 14 displays the experimental scenes used to investigate the estimation accuracy of the calibration-and-estimation model. The estimated Arabic numerals were displayed on the monitor after the subject threw balls toward a target plate determined in advance in the experiments. Through these experiments, we confirmed that the experimental setting and devices worked well and that $R_{a}^{\prime }$ could be estimated using the model.

**Figure 15:**
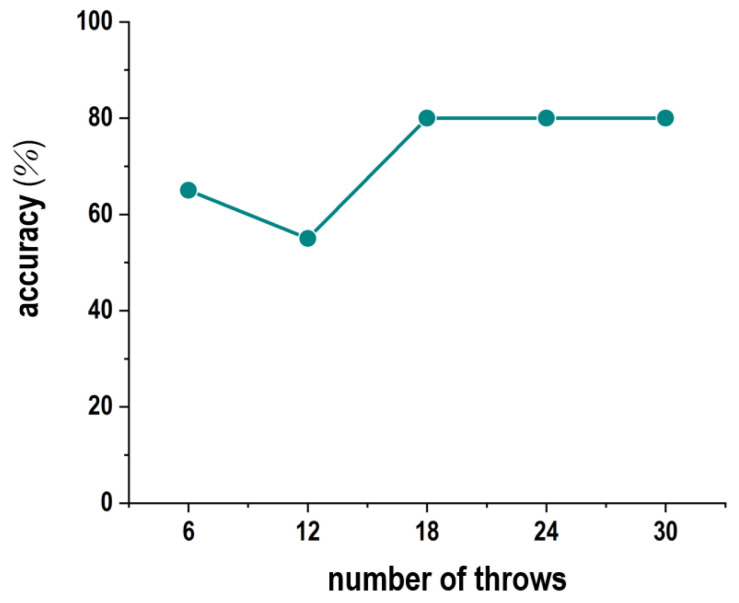
Experimental results for the estimation accuracy according to the number of throws.

Figure 15 presents the experimental results for the estimation accuracy. Here, we prepared five types of experimental settings by changing the numbers of throws in the calibration module stage. In particular, these results indicate the estimation accuracy when using the prediction equation calculated by throwing five balls toward each number plate in advance. Regarding the estimation accuracy when three balls were thrown in advance at each number plate (total of 18 balls), the accuracy remained at about 80% even when the number of balls increased. This outcome was connected to the fact that the prediction formula converged based on the increase in the number of balls thrown. Consequently, with the throwing of approximately 20 balls in advance, the proposed model could perform the estimation with a stable level of accuracy.

**Figure 16:**
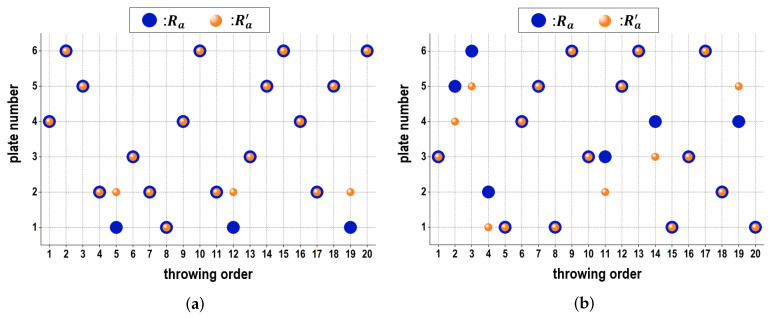
Comparative results between $R_{a}$ and $R_{a}^{\prime }$ according to each throw when subject #2 and subject #3 pitched each of 20 balls, respectively. (**a**) case of subject #2, (**b**) case of subject #3.

Table 1 summarizes the estimation accuracy for three subjects when throwing 20 balls after the number of 18 balls were executed in the calibration module. We confirmed that even subject #3, with the lowest estimation accuracy, was able to achieve a high estimation accuracy of 70%. Figure 16 exhibits comparisons between $R_{a}$ and $R_{a}^{\prime }$ per a ball from the subjects with the highest and lowest estimation accuracies. It can be observed that although $R_{a}$ was different from $R_{a}^{\prime }$, the estimated number was close to the actual number. According to the subjects, these experiments display different estimation accuracies under the proposed model. On the different levels of accuracy depending on subjects, these results confirm that individual differences were taken into consideration during the estimation.

**Figure 17:**
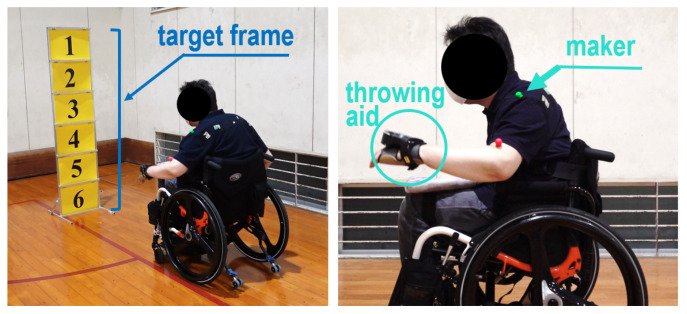
Experiment scene with a boccia player who is unable to grip the ball.

**Figure 18:**
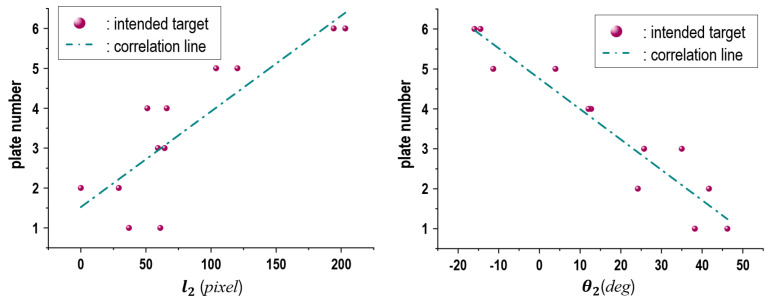
Experimental results for correlations of $l_{2}$ (**left**) and $\theta_{2}$ (**right**) according plate numbers by the boccia player introduced in Figure 17.

Finally, we conducted another experiment with a boccia player who was unable to grip balls. This player threw the ball using a pocket-like brace on the back of the hand (Figure 17). The height of the target frame was adjusted considering the height from his shoulder to the floor. We asked the player to execute throwing motions towards individual numbered plates of the target frame. The player performed the throws twice for each number plate in detail. The obtained results of $l_{2}$ and $\theta_{2}$ are shown in Figure 18. As expected, the results confirm that there was a constant correlation between $l_{2}$ and $\theta_{2}$ and the plates. As a result of these experiments, there seems to be a high possibility that the proposed model is suitable even for people with upper limb disabilities.

The two main features of the calibration and estimation model are described as follows. First, this model focuses only on the movements of the shoulder in the human body during underarm throwing. Second, the model takes individual differences into account during the estimation. In the evaluation tests, we confirmed that by throwing 20 balls in advance the model could account for individual differences in the $R_{a}$ estimation. Consequently, these features could be attributed to the results of movement analysis conducted on multiple subjects. It was verified that a correlation existed between $p_{s}\left(t\right)$ and changes in $R_{a}$ in the movement analyses. Additionally, the slope of the correlation showed variations due to the differences in ball-throwing postures and the rotation range of the shoulder joints. This slope of the prediction equation in a calibration module could be adjusted through the fact that balls were thrown in advance. Therefore, it is possible that the feasibility of a throwing estimation can consider individual differences. However, as our evaluations were limited to a specific age group, we considered studying a wider age group to obtain new insights. In our future work, we will deal with these limitations.

## 5. Conclusions

To estimate ball-throwing intentions based on swinging motions, we determined the shoulder as an appropriate part of the body and investigated its relationship to $R_{a}$ in multiple people. Although there was a correlation in the subjects between $p_{s}\left(t\right)$ and $R_{a}$, individual differences existed in terms of the slope of the correlation. Based on the confirmed relationship, a calibration and estimation model which enabled the consideration of individual differences was proposed, and its effectiveness was proven by conducting extensive experiments.

We expect the proposed model to greatly expand the means of supporting disabled people with ball-throwing disabilities. For example, we plan to develop a ball-throwing robot equipped with the proposed model as an interface. The ball-throwing robot is expected to throw the ball with the same swinging motion as that of a person with ball-throwing disabilities. As this idea does not require an assistant, we aim to spread the joy of participating in sports without the burden of nursing care. In addition, as the launch angle of the ball is controllable, we expect to increase use possibilities from a tactical point of view in boccia matches. As we can control $R_{a}$ during throwing, the robot can enable people with throwing disabilities to intuitively gain enjoyment from the game and improve their physical strength and motor skills in order to realize enhanced sociability.

## Figures and Tables

**Table 1:** Estimation accuracy according to individual subjects when throwing 20 balls.

Subject	Number of Throws	Accuracy (%)
subject #1	20	80
subject #2	20	85
subject #3	20	70

## Data Availability

Data are contained within the article.

## References

[1] Jarvis S., Rainer P., Ganesh S. (2023). Fundamental movement proficiency of Welsh primary school children and the influence of the relative age effect on skill performance—Implications for teaching. Education 3-13.

[2] Hulteen R.M., True T., Kroc E. (2023). Trust the “Process”? When fundamental motor skill scores are reliably unreliable. Meas. Phys. Educ. Exerc. Sci..

[3] Lawson C., Eyre E.L.J., Tallis J., Duncan M.J. (2021). Fundamental movement skill proficiency among British primary school children: Analysis at a behavioral component level. Percept. Mot. Skills.

[4] Vieira I.P., Lobo P.C.B., Fisher J., Ramirez-Campilo R., Pimentel G.D., Gentil P. (2021). Effects of high-speed versus traditional resistance training in older adults. Sport. Health A Multidiscip. Approach.

[5] Jacques C.J., Labbe A., Mercer C., Moran A., Weldy J. (2020). Rehabilitation of the throwing athlete —How to get them back to sport. Oper. Tech. Sport. Med..

[6] Aoyama J.T., Maier P., Servaes S., Serai S.D., Ganley T.J., Potter H.G., Nguyen J.C. (2019). MR imaging of the shoulder in youth baseball players: Anatomy, pathophysiology, and treatment. Clin. Imaging.

[7] Jan Z., Abas M., Khan I., Qazi M.I., Jan Q.M.U. (2024). Design and analysis of wrist hand orthosis for carpal tunnel syndrome using additive manufacturing. J. Eng. Res..

[8] Popescu D., Iacob M.C., Tarba C., Laptoiu D., Cotruţ C.M. (2024). Exploring a novel material and approach in 3D-printed wrist-hand orthoses. J. Manuf. Mater. Process..

[9] Bos R.A., Haarman C.J., Stortelder T., Nizamis K., Herder J.L., Stienen A.H.A., Plettenburg D.H. (2016). A structured overview of trends and technologies used in dynamic hand orthoses. J. Neuroeng. Rehabil..

[10] Chen Z., Min H., Wang D., Xia Z., Sun F., Fang B. (2023). A review of myoelectric control for prosthetic hand manipulation. Biomimetics.

[11] Pascal W., Julia S., Samuel R., Felix H., Tamim A. (2022). Designing prosthetic hands with embodied intelligence: The KIT prosthetic hands. Front. Neurorobotics.

[12] Dunai L., Novak M., García Espert C. (2021). Human hand anatomy-based prosthetic hand. Sensors.

[13] Vertongen J., Kamper D.G., Smit G., Vallery H. (2021). Mechanical aspects of robot hands, active hand orthoses, and prostheses: A comparative review. IEEE/ASME Trans. Mechatronics.

[14] Immaculada L.-H., Perez-Gonzalez A., Veronica G.-I. (2019). Anthropomorphism index of mobility for artificial hands. Appl. Bionics Biomech..

[15] Suarez-Iglesias D., Carlos A.P., Nuria M.-L., Villa-Vicente J.G. (2020). Boccia as a rehabilitation intervention for adults with severe mobility limitations due to neuromuscular and other neurological disorders: Feasibility and effects on upper limb impairments. Front. Psychol..

[16] Koper M., Nadolska A., Urbanski P., Wilski M. (2020). Relationship between pre-competition mental state and sport result of disabled boccia athletes. Int. J. Environ. Res. Public Health.

[17] Suzuki R., Onishi R., Kasamatsu K., Shimomura Y., Nitta O., Motegi R., Tsuchiya S., Shida N., Takesue N., Yamamoto S., Mori H. (2019). Development of boccia robot and its throwing support interface. Human Interface and the Management of Information. Information in Intelligent Systems.

[18] Deng M., Chen R., Song S., He J., Onishi R., Suzuki R., Motegi R., Takesue K., Tsuchiya S., Shimomura Y., Stephanidis C. (2019). Interface design for boccia robot considering operation characteristic. HCI International 2019—Late Breaking Papers.

[19] Gorski F., Grohs A., Kuczko W., Żukowska M., Wichniarek R., Siwiec S., Băilă D.-I., Zelenay M., Păcurar R., Sanfilippo F. (2024). Development and studies of VR-assisted hand therapy using a customized biomechatronic 3D printed orthosis. Electronics.

[20] Nakayama J., Sunagawa K., Ogawa K., Oka H. (2021). Analysis of a new artificial muscle type dynamic orthosis for wrist joint disease using a three-dimensional motion analyzer. Prog. Rehabil. Med..

[21] Yamac G., Chai J.J.K., O’Sullivan C. (2023). Let it go! Point of release prediction for virtual throwing. Comput. Graph..

[22] Reina R., Dominguez-Diez M., Urban T., Roldan A. (2018). Throwing distance constraints regarding kinematics and accuracy in high-level boccia players. Sci. Sport..

[23] Maselli A., Dhawan A., Cesqui B., Russo M., Lacquaniti F., d’Avella A. (2017). Where are you throwing the ball? I better watch your body, Not just your arm!. Front. Hum. Neurosci..

[24] Hendarto S., Doewes R.I., Gontara S.Y., Manshuralhudlori M. (2023). Gender differences in boccia underhand throw biomechanics. Ibero-Am. J. Exerc. Sport. Psychol..

[25] Angel-Lopez J.P., Castellanos-Ruiz J., Rojas-Lopez L.T., Ramirez-Restrepo D., Gomez-Alzate A., Orozco-Ocampo Y.M., Aguirre-Ospina C.A., Hoyos-Restrepo M.C., González Díaz C.A., González C.C., Leber E.L., Vélez H.A., Puente N.P., Flores D.-L., Andrade A.O., Galván H.A., Martínez F., García R. (2020). Boccia assistant biomechanics: A case study. Proceedings of the VIII Latin American Conference on Biomedical Engineering and XLII National Conference on Biomedical Engineering.

[26] Doewes R.I., Nuryadin I., Agustiyanta U., Adirahma A.S. (2020). Analysis of underhand throwing movement on cerebral palsy athletes. Int. J. Adv. Sci. Technol..

[27] Yokota H., Ohshima S., Mizuno N. (2015). Information visualisation of optimised underhand throw for cybernetic training. Procedia Eng..

[28] Ramasamy Y., Usman J., Razman R., Wei Y.M., Towler H., King M. (2023). A systematic review of the biomechanical studies on shoulder kinematics in overhead sporting motions: Types of analysis and approaches. Appl. Sci..

[29] Trasolini N.A., Nicholson K.F., Mylott J., Bullock G.S., Hulburt T.C., Waterman B.R. (2022). Biomechanical analysis of the throwing athlete and its impact on return to sport. Arthrosc. Sport. Med. Rehabil..

[30] Medina G., Bartolozzi A.R., Spencer J.A., Morgan C. (2022). The thrower’s shoulder. J. Bone Jt. Surg. Rev..

[31] Torabi T.P., Juul-Kristensen B., Dam M., Zebis M.K., van den Tillaar R., Bencke J. (2022). Comparison of shoulder kinematics and muscle activation of female elite handball players with and without pain—An explorative cross-sectional study. Front. Sport. Act. Living.

[32] Schwarz A., Bhagubai M.M.C., Nies S.H.G., Held J.P.O., Veltink P.H., Buurke J.H., Luft A.R. (2022). Characterization of stroke-related upper limb motor impairments across various upper limb activities by use of kinematic core set measures. J. Neuroeng. Rehabil..

[33] Mick S., Segas E., Dure L., Halgand C., Benois-Pineau J., Loeb G.E., Cattaert D., de Rugy A. (2021). Shoulder kinematics plus contextual target information enable control of multiple distal joints of a simulated prosthetic arm and hand. J. Neuroeng. Rehabil..

[34] Cross R. (2018). Throwing accuracy. Phys. Educ..

[35] Dupuy M.A., Mottet D., Ripoll H. (2000). The regulation of release parameters in underarm precision throwing. J. Sport. Sci..

[36] Braatz J.H., Gogia P.P. (1987). The mechanics of pitching. J. Orthop. Sport. Phys. Ther..

